# Posttranslational regulation of the GCN5 and PCAF acetyltransferases

**DOI:** 10.1371/journal.pgen.1010352

**Published:** 2022-09-15

**Authors:** Onyekachi E. Ononye, Michael Downey

**Affiliations:** 1 Department of Cellular & Molecular Medicine, University of Ottawa, Ottawa, Ontario, Canada; 2 Ottawa Institute of Systems Biology, University of Ottawa, Ottawa, Ontario, Canada; The University of North Carolina at Chapel Hill, UNITED STATES

## Abstract

General control nonderepressible 5 protein (Gcn5) and its homologs, including p300/CBP-associated factor (PCAF), are lysine acetyltransferases that modify both histone and non-histone proteins using acetyl coenzyme A as a donor substrate. While decades of studies have uncovered a vast network of cellular processes impacted by these acetyltransferases, including gene transcription and metabolism, far less is known about how these enzymes are themselves regulated. In this review, we summarize the type and functions of posttranslational modifications proposed to control Gcn5 in both yeast and human cells. We further outline common themes, open questions, and strategies to guide future work.

## Introduction

Protein lysine acetylation (hereafter acetylation) is a posttranslational modification (PTM) whereby the acetyl group from acetyl coenzyme A (acetyl-CoA) is transferred to the ε-nitrogen of lysine amino acids within target proteins (reviewed in [1]). While acetylation is perhaps best understood as a histone modification with roles in eukaryotic transcription, non-histone proteins are also frequent targets for acetylation in organisms from yeast to humans [2]. For this reason, while the enzymes that catalyze acetylation were first called HATs (standing for histone acetyltransferase), the more inclusive term “KATs” (standing for lysine (K) acetyltransferase) has gained traction. Since the identification of yeast general control nonderepressible 5 protein (Gcn5) as an acetyltransferase in 1996 [3], Gcn5 and its homologs have emerged as arguably the best studied of all KAT enzymes [4].

In the budding yeast *Saccharomyces cerevisiae*, Gcn5 is thought to function exclusively as a member of large protein complexes. Chief among these is the SAGA (Spt-Ada-Gcn5 acetyltransferase) complex, an approximately 20 subunit transactivator machine that can be functionally divided into submodules, including 2 that harbor enzymatic activities [5–7]. First, the KAT submodule contains Gcn5, but also Ada2, Ada3, and Sgf29. Ada2 and Ada3 in particular are important for Gcn5 interaction with SAGA, and Ada2 also promotes Gcn5’s binding to acetyl-CoA [8,9]. Second, the deubiquitylation or “DUB” submodule contains Ubp8 that removes monoubiquitin from histone H2B [10]. Gcn5 also integrates into the related SLIK (SAGA-like) complex, which has a truncated version of the scaffolding protein Spt7 and lacks Spt8 at the expense of Rtg2 [11,12]. These alterations may serve to integrate the function of SLIK with the sensing of mitochondrial dysfunction [11]. Finally, the KAT submodule, along with Ahc1 and Ahc2 proteins, can also exist in a distinct complex called ADA, which is thought to have unique roles in transcription regulation [8].

**Fig 1:**
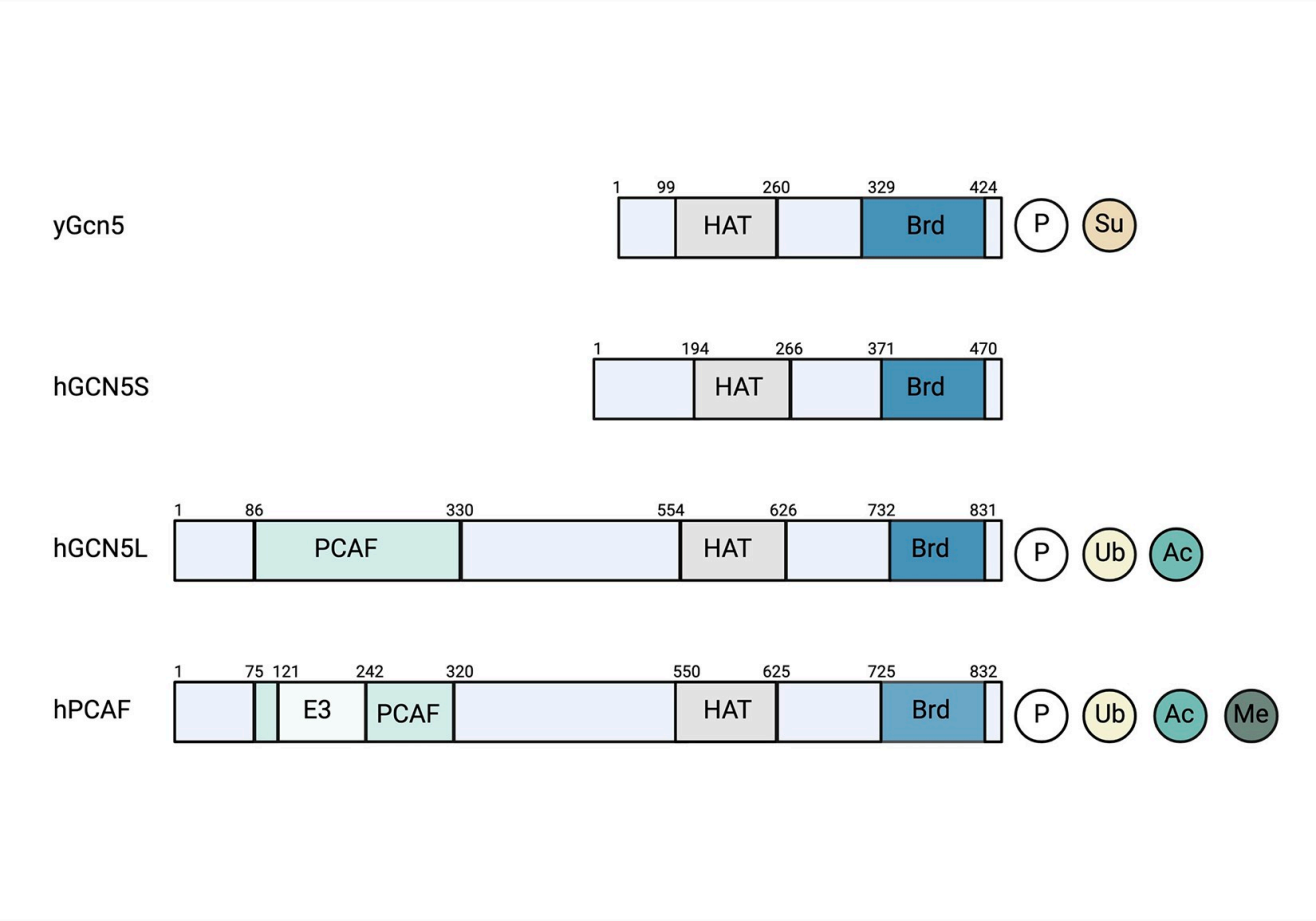
Domain structure of yeast Gcn5 compared to human GCN5 and PCAF homologs. Functional domains of yeast yGcn5 and mammalian hGCN5 and hPCAF outlining E3 ligase, PCAF homology, acetyl transferase (HAT), and bromodomain (Brd) regions with known PTMs: phosphorylation (P), sumoylation (Su), ubiquitylation (Ub), acetylation (Ac), and methylation (M). Created with BioRender.com.

Both Gcn5 and most of the other members of the SAGA complex are highly conserved from yeast to humans [5]. Notably, it has been suggested that there are 2 forms of mammalian GCN5 that derive form a poorly characterized splicing event [13,14]. Although both forms have been detected at the mRNA levels in liver and liver cancer [15], the relative expression of each protein isoform is unclear. The short protein form of GCN5 is similar to yeast Gcn5 (**Fig 1**). The longer form includes an N-terminal region (PCAF homology domain) that is also found in GCN5 paralog PCAF (KAT2B), which can substitute for GCN5 in the SAGA complex (**Fig 1**). Adding to this complexity, both GCN5 and PCAF separately incorporate into a distinct acetyltransferase complex called ATAC (Ada-Two-A-containing), which has unique properties as a transcriptional regulator [5]. Beyond GCN5 or PCAF, ATAC shares ADA3 and SGF29 with the SAGA complex [5]. However, as suggested by its name, ATAC incorporates ADA2A in place of the ADA2B protein found in SAGA, in addition to 6 core ATAC proteins that facilitate interaction with chromatin [5]. The relationship between the structures of SAGA and related complexes and their proposed functions are beyond the scope of this review but are summarized in an excellent article by Helmlinger and colleagues [5]. It is noteworthy that many studies focused on GCN5 and PCAF biology fail to properly consider or discuss their placement within these larger complexes.

While GCN5 and PCAF share many targets and are often discussed together [16], genetic analyses demonstrate that they do indeed have distinct functions. This is perhaps best illustrated by the observation that while mouse *Gcn5* knockouts are embryonically lethal, *Pcaf* knockouts are not, and double knockouts die earlier than single *Gcn5* mutant embryos [17,18]. There is at least one report of *GCN5* and *PCAF* knockdowns having opposite phenotypes in cells grown in culture [19], hinting that the differences go beyond tissue-specific expression, perhaps involving changes in targeting or regulation.

While we have gained considerable insight into the diversity of proteins targeted by Gcn5 [16], we know surprisingly little about how Gcn5 itself is regulated at the posttranslational level. This review will provide insight into this question. We will start by summarizing the regulation and function of known PTMs for yeast Gcn5 and human GCN5 and PCAF. Then, we follow with a discussion of common themes and open questions and end by discussing strategies to guide future work.

We will use the gene and protein nomenclature conventions appropriate for each organism as they are considered. For simplicity, when discussing aspects of Gcn5 biology that apply to multiple organisms, we will stick with the nomenclature used for budding yeast. **Table 1** provides a summary of known PTMs discussed in the text.

**Table 1:** Posttranslational modifications of Gcn5-like acetyltransferases in yeast and mammalian cells.

Protein	Modification	Modifier	Sites	Domain	KAT activity	Cell/tissue used
**yGcn5**	Phosphorylation	Snf1 [23]	T203, S204, T211, and Y212	HAT	Unknown	-
	Sumoylation	Ubc9 as E2 in vitro [52,54]	K25	N-term	Unknown	-
**GCN5**	Phosphorylation	Cyclin D1-CDK4 [34]	T272, S372	PCAF	Increased	U2OS, Hep2, primary hepatocytes
		Protein kinase A (PKA) [35]	S275	PCAF	Increased	Fao cells, primary hepatocyte
		DNA-Protein kinase (DNA-PK) [36]	Unknown	Bromodomain	Decreased	HeLa, M059K
	Ubiquitylation	CRL4^Cdt2^ [56]	Unknown	Partial bromodomain (667–749)	Degraded protein = no activity	HCT116, HeLa, U2OS
	Deacetylation	SIRT6 [63]	K549	HAT	Increased	U2OS, HEK293
**PCAF**	Phosphorylation	ATR [37]	S264	PCAF	Decreased	U2OS, HEK293
		DNA-protein Kinase (DNA-PK) [38]	Unknown	Unknown	Increased	HeLa, HEK293T
		Rsk2 and MSK1/2 [39]	Unknown	HAT	Unknown	Pc-12
	Autoubiquitylation	PCAF [61]	N-term (350–445)	E3	Decreased	In vitro
	Ubiquitylation	MDM2 [58]	N-term	N-term	Degraded protein = no activity	H1299, HEK293
	Autoacetylation	PCAF [66]	Unknown	NLS	Increased	U2OS, C2C12
	Deacetylation	SIRT7 [64]	K720	Bromodomain	Unknown	HCT116, U2OS
	Methylation	Set9 [70]	K78, K79	N-term	Unknown	HEK293, U2OS

## Regulation by phosphorylation

Phosphorylation is a PTM catalyzed by kinases, which covalently add a phosphate moiety to amino acid side chains, principally those of serine, threonine, and tyrosine [20,21]. Phosphorylations can be reversed by phosphatases, which transfer the phosphate group to a water molecule, restoring the amino acid to its original state [22].

In yeast, the glucose-sensing kinase Snf1 phosphorylates Gcn5 on 4 residues in its catalytic domain (T203, S204, T211, and Y212) [23]. Mutation of these 4 residues to alanine (TSTY➔A) drastically reduces Gcn5 phosphorylation, concomitant with decreased transcription of *HIS3*, a SAGA-target gene encoding an enzyme required for histidine biosynthesis [23]. A lack of H3 K14 acetylation at the *HIS3* promoter suggests an impairment of Gcn5 acetyltransferase activity in vivo [23]. Whether this is a direct effect of decreased Gcn5 catalytic activity that also manifests in vitro has not been tested. In isolation, these data point to a straightforward model, where acetylation promotes Gcn5 activity, at least toward histones at some promoters. However, the situation is likely more complex. Specifically, overexpression of Snf1 can rescue the Gcn5 TSTY➔A mutant’s defects in H3 acetylation and *HIS3* transcription [23], which would not be possible if the only role of Snf1 in regulating Gcn5 was via phosphorylation of these sites. Thus, the true role of Snf1 phosphorylation of Gcn5 remains unclear. There is also the important question of how these phosphorylations might be reversed. As suggested previously [23], the Glc7 phosphatase is an interesting candidate based on its known role in regulating Snf1 itself [24]. Notably, Glc7 and its accessory subunits also copurify with Gcn5 in immunoprecipitation experiments [25–28].

Snf1 is itself subject to complex regulation, with different sets of interactors proposed to regulate its activity in response to changing glucose concentrations [29]. As such, we anticipate that Gcn5 phosphorylation will be sensitive to these same environmental changes, although this remains to be tested. Finally, Snf1 is also regulated by Ubp8-dependent deubiquitylation [30], suggesting that the SAGA DUB submodule could impact Gcn5 phosphorylation and downstream effects of this modification. It is also possible that Gcn5 phosphorylation changes its activity toward non-histone substrates. Using acetylome profiling to compare global acetylation changes in wild-type and *snf1*Δ cells expressing Gcn5 or Gcn5 TSTY➔A mutants may be an excellent strategy to clarify the relationship between the 2 proteins.

There are no detailed reports of *S*. *cerevisiae* Gcn5 being regulated by kinases other than Snf1, but we note that kinase Ptk2 (ion transport and spermine uptake) and Pho85 (phosphate signaling) were found to phosphorylate Gcn5 in a high-throughput protein microarray experiment [31]. Since we expect that regulators of Gcn5 will be involved in diverse aspects of nutrient sensing, these kinases represent ideal leads for future studies.

Similar to yeast, human GCN5 is known for its role in regulating gene expression, as well as serving as a nutrient sensor and regulator of cellular metabolism, activities which are impacted by phosphorylation [32]. Of particular importance is gluconeogenesis—the de novo synthesis of glucose from non-carbohydrate molecules [33]. When insulin levels are high, cyclin D1 accumulates and forms a complex with the CDK4 kinase [34]. This complex directly interacts with GCN5, phosphorylating it at T272 and S372 in its PCAF homology domain. The result is a cell-cycle independent increase in acetyltransferase activity [34]. In contrast, in a fasted state, GCN5 is phosphorylated at S275 by protein kinase A (PKA) following its interaction with transcriptional co-regulator CITED2 [35]. While phosphorylation by CDK4 and PKA both impact GCN5 activity, the end result—or at least the characterized one—is different. CDK4 phosphorylation of GCN5 results in increased acetylation of transcriptional co-activator PGC-1α, which negatively regulates its function thereby preventing the up-regulation of gluconeogenic genes [34]. On the other hand, phosphorylation by PKA induces a change in GCN5’s substrate targeting to favor histone H3 instead of PGC-1α. The resulting hypoacetylation of PGC-1α activates it, triggering gluconeogenesis [35]. We speculate that these type of phosphoswitches may be a common way to regulate GCN5 function in response to changing environmental conditions. Notably, phosphorylation can also have an inhibitory effect on GCN5 activities. Work by Barlev and colleagues indicates that phosphorylation of the GCN5 bromodomain by the DNA protein kinase (DNA PK) holoenzyme, which binds to GCN5 via the Ku70 protein, induces a decrease in its acetyltransferase activity [36]. Although the functional consequences are unclear, this study is noteworthy as it was one of the first describing the posttranslational regulation of GCN5.

PCAF is also directly regulated by phosphorylation. Kim and colleagues revealed that during hydroxyurea-induced inhibition of DNA replication, PCAF is phosphorylated by the ATR kinase at S264 within its PCAF homology domain [37]. This prevents further damage to stalled replication forks by inhibiting PCAF accumulation and hyperactivation, and ultimately, recruitment of the MRE11 and EXO1 nucleases. During UV damage, however, DNA PK is proposed to phosphorylate PCAF to promote its autoacetylation. This leads to downstream acetylation of the single-stranded DNA-binding protein RPA1, a critical regulator of DNA repair [38]. It is not known which residues on PCAF are modified after UV treatment. However, we speculate that in response to different types of DNA damage, unique phosphorylation signatures might direct the KAT toward specific types of DNA repair. Finally, phosphorylation also regulates PCAF’s interactions with the p53 tumor suppressor. During neuronal differentiation, PCAF is phosphorylated within its KAT domain by calcium-dependent kinases Rsk2 and MSK1/2. This is thought to promote localization of PCAF to the nucleus where it acetylates p53 [39]. The serine/threonine kinase HIPK2 also impacts PCAF localization upstream of p53 acetylation, but whether HIPK2 directly phosphorylates PCAF is unclear [40]. Clearly, we are only scratching the surface when it comes to understanding phospho-regulation of Gcn5 and its homologs.

## Regulation by ubiquitylation and ubiquitin-like modifiers

Ubiquitylation is a reversible PTM that covalently links the glycine residue at the carboxyl terminus of the 8.6 kDa ubiquitin protein to target lysine residues, and less frequently cysteines or other amino acids, on target proteins [41–44]. This process involves a series of enzymes referred to as E1, E2, and E3, which activate ubiquitin, transfer, and then ligate it to the protein target, respectively [43,45]. In some cases, this can be reversed by deubiquitylating enzymes (DUBs) that remove ubiquitin from protein targets [46]. Ubiquitin itself can also be ubiquitylated at various lysine residues to form chains. These chains can direct the target protein to the proteasome for degradation or serve to regulate protein–protein interactions [47].

In yeast, little is known about Gcn5 regulation by the ubiquitin proteosome system, although Turner and colleagues reported that Gcn5 turnover in cycloheximide chase assays depends on a large E3 complex called the anaphase-promoting complex (APC) during G1 phase of the cell cycle [48]. The relevant sites of ubiquitylation on Gcn5 are unknown and finding these will be an important step in understanding the functional importance of Gcn5 turnover. It will also be useful to determine where Gcn5 ubiquitylation occurs within the cell. For example, targeted degradation of Gcn5 at select promoters could be one way to fine-tune histone acetylation and transcription. In some circumstances, the proteasome could have a positive effect on Gcn5 activity. For example, the ATPase activity of the proteasomal 19s regulatory particle increases Gcn5-dependent H3 acetylation levels by promoting SAGA recruitment to target genes [49]. However, more work is required to understand how this function might be balanced with Gcn5 turnover. Finally, it is intriguing that Gcn5 itself controls the stability of the nuclear proteins involved in chromatin regulation and DNA repair by directing them toward degradation by the autophagy system [50,51]. We speculate that Gcn5 regulation by the APC or other ubiquitin ligases may provide a link between the ubiquitylation and autophagy systems during cellular stress.

Ubiquitin itself is part of a larger family of ubiquitin-like modifiers that are conserved throughout evolution. This family includes the small ubiquitin-like modifier (SUMO), which has its own set of E1 to E3 enzymes. In 2006, Sterner and colleagues showed that yeast Gcn5 is sumoylated, with lysine 25 being the predominate site [52]. This finding is supported by more recent work by Ng and colleagues, who found other subunits of SAGA complex are also sumoylated in addition to Gcn5 [53]. The enzymes involved in regulation of Gcn5 sumoylation in vivo are unknown, although Ubc9 E2, involved in transcriptional regulation, can sumoylate Gcn5 in vitro [54]. It is noteworthy that only a small fraction of Gcn5 accumulates as a sumoylated form in western blotting experiments [53]. This pattern may reflect rapid sumoylation and desumoylation cycles and modification of a subset of Gcn5, such as that associated with promoters. What then is the function of Gcn5 sumoylation? Although Gcn5 sumoylation was decreased in cells grown in a non-fermentable carbon source, cells expressing Gcn5 with lysine 25 mutated to arginine as their only source of Gcn5 did not have any obvious phenotypes [52]. However, fusion of a SUMO moiety to the N-terminus of the protein, mimicking constitutive sumoylation, resulted in sensitivity to amino acid starvation and decreased transcription of the SAGA-regulated *TRP3* gene [52]. To our knowledge, there have been no attempts to further probe the functions of this modification since the work of Sterner and colleagues in 2006, and this area remains ripe for investigation.

Given that both yeast and human GCN5 exist in different protein complexes, selective degradation of GCN5 may play a role in dictating the biogenesis and destruction of these complexes. In humans, one of these complexes is formed by histone H3 and And-1, a high mobility group DNA-binding protein that stabilizes GCN5 to promote acetylation of H3 K9 and H3 K56 [55]. In the absence of And-1, GCN5 is polyubiquitylated and degraded by the proteasome, resulting in decreased histone acetylation. The mechanism at play is an interesting one: And1 binding to GCN5 at a region overlapping with its bromodomain, prevents GCN5’s binding to the CRL4^Cdt2^ ubiquitin ligase complex that targets it for degradation [56]. Intriguingly, a recent report suggests the serine/threonine kinase Akt1 plays a role in the binding of CRL4^Cdt2^ to GCN5 to promote its degradation in mouse embryonic fibroblasts, although more work is required to outline this mechanism [57]. It would be intriguing to test whether this occurs via disruption of the GCN5–And-1 interaction, as And-1 levels are elevated in mouse embryonic fibroblasts deficient in Akt1 [57].

PCAF is regulated by ubiquitination at its N-terminus via the direct interaction with the E3 ubiquitin ligase, MDM2 [58]. This regulatory mechanism was first speculated when Jin and colleagues discovered that MDM2 inhibited PCAF-dependent acetylation of p53 [59]. It was established that ubiquitylation of PCAF diminishes its half-life and ultimately leads to the degradation of the protein. Interestingly, PCAF itself has been shown to possess E3 ubiquitin ligase activity within its N-terminus, even though it does not share homology with other known E3 ligases [60]. PCAF undergoes autoubiquitylation between residues 350 to 445, and, in vitro, this inhibits the PCAF-dependent acetylation of p53 [61]. More recently, Toma-Fukai and colleagues found that GCN5 can also autoubiquitylate within its PCAF domain, although the impact of this modification is unknown [60]. In future work, it will be imperative to identify cellular conditions promoting degradation of these acetyltransferases and to find which DUBs are responsible for the reverse reaction. Potential regulators could be found within the SAGA “DUB” submodule that contains Ubp8 (yeast) and USP22 (human) [62].

## Regulation by acetylation and deacetylation

Protein lysine acetylation is often reversed by lysine deacetylases (KDACs) that remove acetyl groups from target residues. Previous studies have revealed that the activities of both GCN5 and PCAF are impacted by KDACs. For example, the co-expression of both GCN5 and the deacetylase, sirtuin 6 (SIRT6), led to an increase in in vitro KAT activity, concomitant with the loss of the K549 acetylation on GCN5 [63]. While the KAT that causes this acetylation is unknown, it was suggested that loss of the modification, located in the protein’s KAT domain, induced structural changes that enhance its acetyltransferase activity. Interestingly, mass spectrometry work from this group revealed that 2 sites were phosphorylated (S307 and T735) following the deacetylation event [63]. This suggests an additional layer of regulation wherein changes to one modification impacts others on the same substrate.

PCAF, on the other hand, directly interacts with the deacetylase sirtuin 7 (SIRT7) during glucose deprivation, which results in PCAF deacetylation at K720 [64]. Deacetylation promotes PCAF’s binding to MDM2, followed by degradation of MDM2 in a manner dependent on PCAF’s E3 ubiquitin ligase activity [64]. Thus, while MDM2 can regulate the stability of PCAF (see “Regulation by ubiquitylation and ubiquitin-like modifiers”), the reverse is also true, and this might be regulated by the acetylation status of PCAF.

As mentioned above, while KATs are known to acetylate other proteins, they are also able to undergo autoacetylation [65]. PCAF autoacetylation is particularly interesting as it occurs on the protein’s N terminus within a region (aa 425 to 445) known to carry its nuclear localization signal (NLS), and autoacetylation is thought to promote PCAF accumulation in the nucleus [66]. PCAF also demonstrated increased acetylation of histone H3 when autoacetylated. Although autoacetylation of GCN5 has not been investigated in detail, it is unknown if the acetylation event on GCN5 (K549) discussed earlier is due to autoacetylation or modification by other KATs [63].

There is little known about regulation of yeast Gcn5 acetylation, although a number of acetylation sites on Gcn5 have been mapped [67]. Here again, it seems likely that many of these could be sites of autoacetylation. Indeed, multiple members of the SAGA complex are regulated by Gcn5-dependent acetylation. This includes Ada3, whose acetylation by Gcn5 is important for SAGA dimerization [68].

## Regulation by methylation

Methylation is the addition of a methyl group to select amino acids (arginine, proline, lysine, histidine) on histone and non-histone proteins by methyl transferases [69]. Masatsugu and colleagues demonstrated that the methyl transferase Set9 can mono-methylate PCAF, both in vitro and in vivo, primarily at K78 and K89 [70]. While nothing is known about their functions, these methylations are intriguing because they could block other lysine-based PTMs such as acetylation and ubiquitylation. In addition to examining the function of these sites, it will be important for future studies to determine if other methyltransferases can modify PCAF, GCN5, or both.

## Current perspectives and open questions

Our knowledge of how PTMs on Gcn5 proteins regulate their functions is fragmentary at best. While the current literature provides us with a collection of interesting examples, there has been little effort invested into understanding how various modifications work together to direct Gcn5’s activities in time and space. The coordination of various PTMs may be particularly important in highly modified regions. For example, N-terminal region of PCAF and the PCAF homology domain in GCN5 appear to be frequently modified, and this region could serve as a hub for integrating signals in response to changing environmental conditions. As outlined below, we suggest that a broad “systems-level” investigation of GCN5 regulation will be critical to moving the field forward.

## Finding new regulators

It would be naïve to assume we have identified all known regulators of Gcn5 proteins or even the most important ones. But where else can we look? We suggest that exploiting existing databases of protein–protein interactions (e.g., BioGRID [28]) may be an excellent place to start. For example, beyond Snf1, at least 6 additional kinases interact with yeast Gcn5 in high-throughput studies, including Cmk1, casein kinase, Ire1, Cla4, Rck1, Psk1 [26,71]. As these kinases are involved in sensing and responding to diverse stresses, they represent exciting points for future investigations. Gcn5 also shows physical interactions with other ubiquitin ligases such as the SCF, Bul1, the Ubp12 DUB, and subunits of the proteasome [26,72]. Interestingly, Gcn5 also interacts with Cdc48 [26], an ATPase that functions in part to separate ubiquitylated/sumoylated substrates from chromatin ahead of degradation by the proteasome [73]. Cdc48 is in an ideal position to participate in the turnover of Gcn5 and/or SAGA-like complexes at select promoters as they are turned off. Notably, interactions between Gcn5 and its modifiers may be only transient in nature. As such, the of use of proximity labeling techniques such as BioID [74] may be particularly useful in identifying these proteins. Moreover, examining how these interactions change in response to different cellular stress may provide new insights into PTM cooperation. As we strive to identify new regulatory mechanisms for Gcn5, it is important to remember that these do not happen in isolation—it is likely a mistake to view any PTM or group of PTMs as isolated events. Instead, akin to the idea of the “histone-code” [75], distinct combinations of PTMs could direct Gcn5 toward specific targets and downstream functions.

## Determining the function of PTMs

**Fig 2:**
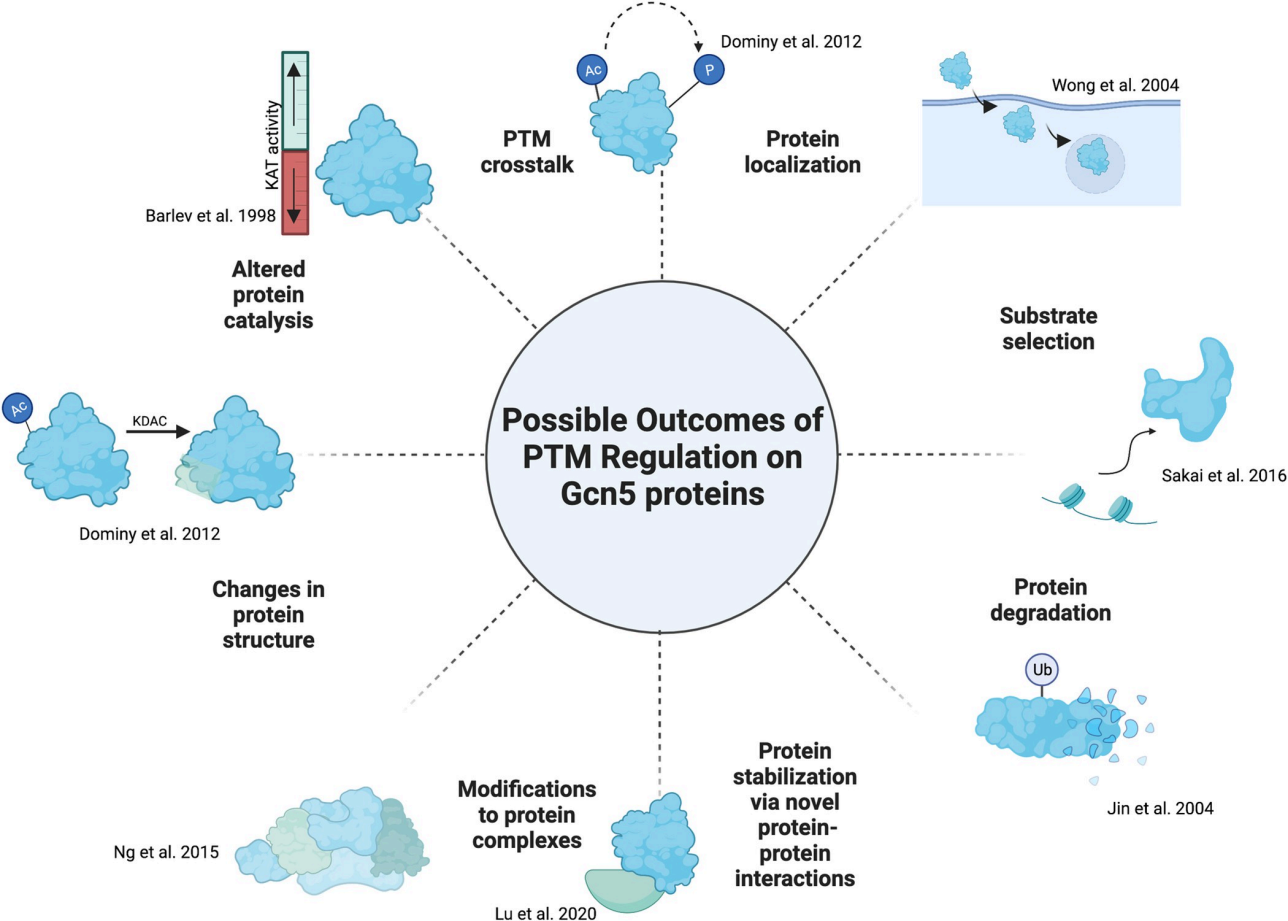
Molecular consequences for the modification of Gcn5 proteins. Overview of cellular processes impacted by the modulation of Gcn5 proteins activities. For details, see indicated studies [35,36,39,53,58,63,64]. Adapted from “Circular Diagram with 8 Sections (Layout)”, by BioRender.com (2022). Retrieved from https://app.biorender.com/biorender-templates.

Finding the function of modifications on any protein can be a long process, even after the writers and erasers of that modification have been identified. We can think about function at 2 levels. The first level focuses on Gcn5 itself. As outlined in **Fig 2**, there are multiple ways that we can envision modifications direct Gcn5 molecules to unique fates and examples of these have been discussed throughout this review. Largely missing from current work is whether Gcn5 PTMs differ when Gcn5 is incorporated into its various complexes (e.g., SAGA, ADA, ATAC). It is tempting to speculate that posttranslational modification of Gcn5 proteins could specify their incorporation into one complex over another, perhaps tilting the balance of their functions during specific stresses, points in the cell cycle, or at different stages of development. The balance of Gcn5 modifications might also be impacted by overexpression of the KAT. Gcn5 that fails to undergo “necessary” posttranslational modifications when overexpressed could impose a dominant or dominant negative effect when incorporated into SAGA-like complexes. Since overexpression of human GCN5 has been proposed as a driver of cancer [76], this could have important consequences for our understanding of this and other disease states.

More challenging is the second level of function, which focuses on the consequences for the cell, downstream of the direct effects on Gcn5 itself. Previous work has focused on specific targets and downstream pathways in isolation. Instead, we encourage the prioritization of studies that take a bird’s eye view of Gcn5 function. With hundreds of potential Gcn5 targets (including both histone and non-histone substrates), acetylome profiling of cells expressing Gcn5 mutants that cannot be posttranslationally modified will allow us to better understand how specific pathways signal through Gcn5, perhaps to alter a subset of its modifications. Given that Gcn5 proteins can also use other acyl-CoA molecules to perform modifications such as crotonylation [77] and succinylation [78], it will be important to test how various PTMs regulate the balance of these activities toward histones and other targets in both yeast and human systems. In parallel, genome-wide genetic interaction screens (i.e., using Synthetic Genetic Array analysis [79] or CRISPR [80]) can be used to better understand the function of modifications. The identification of mutations or knockdowns that display synthetic lethal phenotypes in combination with Gcn5 that cannot be modified (e.g., S-A mutations for phosphorylation or K-R mutants for sumoylation) will be particularly useful in identifying functions for modifications that might work in concert with other signaling pathways. Of course, there is no guarantee that any single PTM impacts the function of its target in a meaningful way. Analyzing Gcn5 mutated for many PTM sites at once may be necessary to mitigate the risk associated with these types of large-scale experiments. The contribution of individual sites can then be deconvolved once phenotypes have been identified to fine-tune our understanding of Gcn5 regulation. Another strategy to mitigate risk may be to embark on a “phenotype-first” approach. Here, saturated site mutagenesis may be useful to pinpoint those amino acids that are, perhaps unexpectedly, important for the regulation of Gcn5 activities.

## Conclusion

While the roles that Gcn5 and its homologs play in modulating cellular protein biochemistry have been widely studied, further exploratory work is necessary to understand the complex regulatory network of PTMs that impinge upon Gcn5 itself. We are confident that these discoveries will provide a better framework for understanding the true breadth of KAT functions across diverse eukaryotic species.
